# Clinical value and potential mechanisms of BUB1B up-regulation in nasopharyngeal carcinoma

**DOI:** 10.1186/s12920-022-01412-8

**Published:** 2022-12-28

**Authors:** Li-Ting Qin, Si-Wei Huang, Zhi-Guang Huang, Yi-Wu Dang, Ye-Ying Fang, Juan He, Yi-Tong Niu, Cai-Xing Lin, Ji-Yun Wu, Zhu-Xin Wei

**Affiliations:** 1 Department of Pathology, First Affiliated Hospital of Guangxi Medical University, Guangxi Zhuang Autonomous Region, 6 Shuangyong Road, Nanning, 530021 People’s Republic of China; 2 Department of Radiotherapy, First Affiliated Hospital of Guangxi Medical University, Guangxi Zhuang Autonomous Region, 6 Shuangyong Road, Nanning, 530021 People’s Republic of China

**Keywords:** Nasopharyngeal carcinoma (NPC), BUB1 mitotic checkpoint serine/threonine kinase B (BUB1B), Standard mean deviation (SMD), Immunohistochemical (IHC)

## Abstract

Nasopharyngeal carcinoma (NPC) has insidious onset, late clinical diagnosis and high recurrence rate, which leads to poor quality of patient life. Therefore, it is necessary to further explore the pathogenesis and therapy targets of NPC. BUB1 mitotic checkpoint serine/threonine kinase B (BUB1B) was found to be up-regulated in a variety of cancers, but only two previous study showed that BUB1B was overexpressed in NPC and the sample size was small. The clinical role of BUB1B expression and its underlying mechanism in NPC require more in-depth research. Immunohistochemical samples and public RNA-seq data indicated that BUB1B protein and mRNA expression levels were up-regulated in NPC, and summary receiver operating characteristic curve indicated that BUB1B expression level had a strong ability to distinguish NPC tissues from non-NPC tissues. Gene ontology and Kyoto Encyclopedia of genes and genomes were performed and revealed that BUB1B and its related genes were mainly involved in cell cycle and DNA replication. Protein- Protein Interaction were built to interpret the BUB1B molecular mechanism. Histone deacetylase 2 (HDAC2) could be the upstream regulation factor of BUB1B, which was verified by Chromatin Immunoprecipitation Sequencing samples. In summary, BUB1B was highly expressed in NPC, and HDAC2 may affect cell cycle by regulating BUB1B to promote cancer progression.

**Supplementary Information:**

The online version contains supplementary material available at 10.1186/s12920-022-01412-8.

## Introduction

Budding uninhibited by benzimidazoles 1 homolog β (BUB1B), located at 15q15-1, belongs to the family of the spindle assembly checkpoint (SAC), encodes mitotic checkpoint serine/threonine kinase B and involves in the function of spindle checkpoint and chromosome segregation. The protein is located in the centromere and plays a role in inhibiting anaphase-promoting complex/cyclosome, delaying anaphase and ensuring proper chromosome separation [1, 2]. Spindle checkpoint dysfunction has been found in many forms of cancer [3]. A large number of reports have indicated that overexpression of BUB1B is associated with the progression and prognostic of ovarian cancer, hepatocellular carcinoma, prostate cancer, breast cancer and other cancers [4–7]. Nevertheless, the influence of BUB1B expression on the occurrence and progression of nasopharyngeal carcinoma (NPC) is still unclear.

NPC is an epithelial cancer that arising from the nasopharyngeal mucosa. It has obvious geographic distribution characteristics and is particularly prevalent in East Asia and Southeast Asia. The epidemiological trend in the past decades has shown that its morbidity and mortality have gradually decreased [8, 9]. However, the patient’s quality of life is greatly affected because of the insidious onset of the disease, late clinical diagnosis and high recurrence rate [10]. The molecular mechanisms of NPC pathogenesis have not yet been clarified, which also restricts the further improvement on the treatment level. Therefore, more researches are needed to clarify the pathogenesis of NPC and find the effective therapeutic targets. There has been only two study on the expression of BUB1B in NPC. Huang et al. demonstrated that BUB1B was up-regulated in 22 cases of NPC tissues as compared to 10 non-NPC controls by immunohistochemistry [11]. The number of cases was limited in Huang et al.’s study, and the samples were recruited from a single institute, also only limited approaches were used. Yue et al. confirmed that BUB1B could be a key target for promoting NPC metastasis through bioinformatics tools, but only two GEO data sets were used in the study with a small sample size [12].Further, no comprehensive analysis has been conducted to illustrate the clinical role of BUB1B expression and to explore its underlying mechanism in NPC by far.

Here, we searched the data sets from different high throughput sources, including Gene Expression Omnibus (GEO), Sequence Read Archive (SRA), ArrayExpress, Oncomine, The Cancer Genome Atlas (TCGA) and the literatures that contained the expression pattern of BUB1B in NPC, and conducted a systematic investigation into the collected information. In-house immunohistochemistry (IHC) was applied to test the expression level of BUB1B in clinical NPC samples. Furthermore, the potential regulatory mechanism of BUB1B in NPC was analyzed. Meanwhile, the upstream regulatory factor of BUB1B were also predicted. Based on the strategy of multi-method and multi-sample, we attempted to reveal the key role of BUB1B in NPC. The overall design of this study is presented in Additional file 1: Fig. S1.

## Materials and methods

### High throughput data mining

Microarray or RNA-seq datasets, which could be used to identify BUB1B expression patterns in NPC, were downloaded and extracted from GEO, SRA, ArrayExpress, Oncomine, and publish literature. The inclusion criteria were as follows: (1) All samples must be from human nasopharyngeal tissues, (2) Gene chips or RNA-seq datasets must contain profile measurement of mRNA expression, (3) RNA-seq dataset from the same platform included no less than 3 non-cancerous nasopharyngeal and NPC samples in total, and (4) Each sample must be an original sample from an untreated patient or an untreated cell strain. The data filtering process is shown in Additional file 1: Fig. S2.

### Data preprocessing

To remove the influence of sequencing depth and gene length on the expression levels, the per kilobase of exon model per million reads mapped fragments (FPKM) was converted into transcript per million (TPM) data in the R programming language (version 4.0.2). The DNA annotation data were checked carefully, and non-standardized mRNA expression values were transformed by log2. Data sets from the same platform are collated and merged, and named by the platform name. In addition, the limma voom package and sva package in R 4.0.2 were used to eliminate batch effects between studies.

### Statistical analysis of BUB1B expression pattern in NPC

After preprocessing and extracting the BUB1B mRNA expression data, the BUB1B expression level of each data set was visualized by a violin figure. Subsequently, we processed the expression data of BUB1B in groups of NPC and non-tumor tissues, and expressed them as mean ± Standard deviation (SD), which were obtained by independent sample t test in IBM SPSS Statistics version 23.0. This helped to calculate the standard mean deviation using Stata 15.0. BUB1B mRNA expression cohort was integrated with in-house immunohistochemical data for subgroup analysis. I^2^ test was performed to analyze the heterogeneity of the research. I^2^ > 50% or *p* value < 0.05 indicated the heterogeneity of the study, a random-effects model was used for analysis, otherwise, a common effects model was chosen. To ensure the stability of the results, the effect of each dataset was comprehensively evaluated using sensitivity analysis. In addition, Begg’s trial was used to detect publication bias. There is no obvious publication bias if *p* > 0.05. The receiver operating characteristic curve (ROC) was drawn using GraphPad Prism 8.4, and the area under the curve (AUC) was calculated for exploring the clinical capacity of BUB1B in differentiating NPC from non-NPC samples. AUC values less than 0.7, between 0.7 and 0.9 and greater than 0.9 represented the BUB1B distinguishing ability between NPC samples and the control group were weak, medium and strong, respectively. We calculated the true-positive, false-positive, true-negative and false-negative rates, and determined the cutoff value. Using Stata 15.0, a summary receiver operating characteristic curve (sROC) was drawn to check the general discriminating ability of BUB1B between NPC patients and the control samples. The significance of the sROC AUC value was consistent with the ROC curve. Meanwhile, the sensitivity, specificity and Fagan’s Nomogram were calculated to accurately detect the accuracy and effectiveness of BUB1B in identifying NPC patients from non-neoplasm samples [13–15].

### In-house immunohistochemical staining and evaluation

The tissue microarrays (TMAs) were prepared from 98 NPC and 33 chronic nasopharyngeal mucositis tissue specimens (NPC131, NPC241 and NPC482) by Pantomics, Inc (Richmond, CA). Meanwhile, we also collected 26 clinical tissue specimens consisting of 12 cases of NPC tissues and 14 cases of chronic nasopharyngeal mucositis tissues at First Affiliated Hospital of Guangxi Medical University. The study was approved by the Ethical Committee of the First Affiliated Hospital of Guangxi Medical University. BUB1B protein expression was detected by immunohistochemistry. The nasopharyngeal tissue samples were fixed with formalin and embedded in paraffin, then made into 4 μm-thick tissue sections and deparaffinized subsequently. The antigen recovery was accomplished by pressure cooking at 95 °C for 3 min. The samples were incubated with the first BUB1B rabbit polyclonal antibody diluted 1:500 (Abcam) at 37 °C for 1 h. The remaining immunohistochemistry procedures were carried out in accordance with the manufacturer’s instructions, and the final results were independently estimated by two pathologists (Qin LT and He J). Immunoreactivity score (IRS) was used to research the regional differences in staining. Ten typical high-power fields were randomly observed under the light microscope. The staining intensity and the percentage of stained cells in each sample were evaluating to calculate the final IRS. If there was no staining, the coloring intensity registered as 0, 1 for low staining, 2 for moderate staining and 3 for strong color. Simultaneously, if no cells were stained, the percentage of stained cells reported as 0; for < 10% stained cells, record as 1; 2 for 11–50% stained cells; 3 for 51–80% stained cells; 4 for over 80% stained cells. The above two scores were multiplied to generate an IRS ranged from 0 to 12. The independent sample t test in IBM SPSS Statistics version 23.0 was applied to calculate the IRS of each group.

### Potential clinical significance of BUB1B

To explore the relationship between BUB1B expression level and clinical parameters of NPC patients, we collected and integrated the clinical information of NPC patients in the included dataset, including age, gender, stage, and survival time. The SMD calculation was used to evaluate whether BUB1B expression was different in NPC patients in different age groups (< 60 years old vs. ≥ 60 years old), sex (male vs. female) and stage groups (I/II stage vs. III/IV stage). The Graphpad prism8.0 was used for survival analysis to evaluate the difference in prognosis of NPC patients with different BUB1B expression levels. For internal samples, t test was used to verify whether the BUB1B protein expression levels were different among gender and age groups in NPC patients.

### Screening dis-regulated genes and BUB1B co-expressed genes in NPC

We used the Bioconductor limma software package in the R software environment to preliminary screen up-regulated and down-regulated differentially expressed genes from all included high throughput datasets. The early screening of DEGs standards were as follows: (1) |log2FoldChange| > 1 and (2) adjusted *p* value < 0.05. Next, we calculated the standard mean deviation (SMD) of these gene expression levels to obtain more reliable differential genes based on the 95% confidence interval (CI) range. The Pearson correlation algorithm in the R software environment was used to screen the co-expressed genes of BUB1B in all included expression matrices. The co-expressed genes standards were as follows: (1)|correlation coefficient|≥0.3, (2) *p* value < 0.05. The co-expressed genes were grouped according to the sign of *r* value. We performed a ranking algorithm on gene sets, and selected DEGs and CEGs with a repetition number ≥ 5 for intersection operation. The up-regulated DEGs and co-expressed genes with + *r* values ​​were intersected (gene set A), and the down-regulated DEGs and co-expressed genes with − *r* values also were intersected ​​(gene set B).

### The molecular mechanisms of BUB1B inducing NPC

Intersectional genes were organized to perform functional enrichment to explore the potential mechanisms of BUB1B underlying NPC. R clusterProfiler software package was applied for Gene Ontology (GO) and Kyoto Encyclopedia of Genes and Genomes (KEGG) pathway analysis [16–18]. We imported the genes of the first three KEGG pathways of gene set A and gene set B into STRING (https://string-db.org/), performed protein-protein interaction (PPI) analysis and output TSV files. The files were import into Cytoscape 3.8.1, and the degree method of the cytohubba module was used to automatically calculate and screen out the top hub genes with the strongest protein interaction. The correlation between hub genes was displayed by STRING.

### Screening and analysis of BUB1B upstream transcription factors

We applied the Cistrome Data Browser. Toolkit (http://dbtoolkit.cistrome.org/) to predict the upstream genes [19], which could regulate BUB1B. This toolkit would exhibit a list of the transcription factors that are most likely to regulate BUB1B. The RP score was calculated by the BETA algorithm of Cistrome DB [20]. To identify the potential target genes of HDAC2, we used the analysis samples from the Cistrome Data Browser (http://cistrome.org/db/#/). Due to the lack of NPC-related data sets, the epithelial cell related HDAC2 ChIP-Seq data sets were used to explore potential HDAC2 target genes. To make a visual display of the results, we used Integrative Genomics Viewer (IGV) to analyze and display the chip-seq information of the BUB1B gene. In order to further obtain the expression status of HDAC2 and its relationship with BUB1B, we extracted the expression value of HDAC2 in the public data set to calculate SMD value and sROC curves. The correlation coefficient between BUB1B and HDAC2 was calculated and visualized through GraphPad Prism8.4. In addition, 500 bp base sequences upstream of the BUB1B transcription start site were obtained from UCSC (http://genome.ucsc.edu/index.html). Since the correlation coefficient r and standard error (se) cannot be used directly to merge, we converted the correlation coefficient into Fisher’s Z, then calculated the se of Z. We analyzed and merged through Z and se. Finally, the combined results were converted into correlation coefficients.

## Results

### BUB1B up-regulation in NPC using a comprehensive analysis

**Fig. 1:**
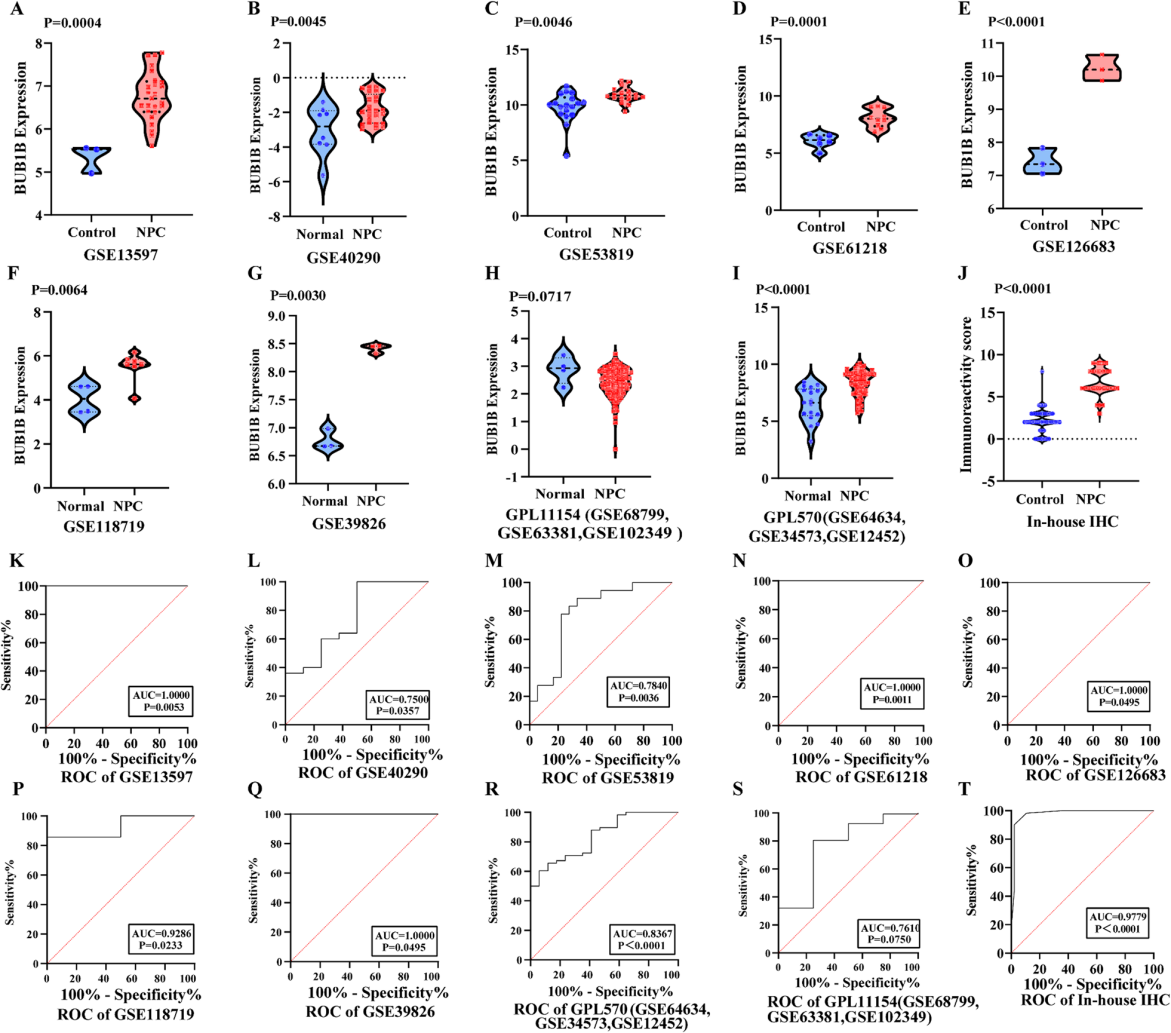
BUB1B expression levels and operating characteristic curves of the BUB1B mRNA expression in NPC tissues based on multiple cohorts. **A**–**I** Different expression levels of BUB1B between NPC and non-tumor nasopharynx tissues based on nine datasets. **J** Different immunoreactivity score between NPC and non-tumor nasopharynx tissues based on in-house samples. **K**–**S** Operating characteristic curves of BUB1B mRNA expression for the distinction of nasopharynx carcinoma from non-tumor tissues based on nine datasets. **T** Operating characteristic curves of BUB1B protein expression for the differentiation of nasopharynx carcinoma from non-tumor tissues based on in-house IHC.

An independent cohort containing BUB1B mRNA expression data from original NPC samples was searched, and 13 gene chips were obtained (Table 1). GSE68799, GSE63381, and GSE102349 were combined into a queue named GPL11154, while GSE64634, GSE34573, and GSE12452 were combined into a cohort called GPL570. The 13 microarrays contained 308 NPC samples and 66 non-tumor nasopharynx tissues. We extracted the expression value of BUB1B in each gene chip and created a violin diagram with scatter points (Fig. 1A–I). The eight of nine datasets showed higher BUB1B mRNA expression in NPC patients than in non-cancerous samples. As for the ROC curve, the data of the 8 datasets showed that the area under the curve value was greater than 0.75 (*p* < 0.05, Fig. 1K–S).

**Table 1:** Characteristics of high throughput datasets and in-house immunohistochemistry (IHC) included in the study

Study	Country	Platforms	Cancer group	Control group	Mean1± SD1	Mean0± SD0	T	*p*
GPL11154(GSE68799, GSE63381, GSE102349)	China, Singapo-re, USA	GPL11154	159	4	2.42 ± 0.49	2.87 ± 0.49	− 1.81	0.07
GPL570(GSE64634, GSE34573, GSE12452)	China, UK, USA	GPL570	58	17	8.30 ± 1.12	6.50 ± 1.51	5.37	< 0.01
GSE118719	USA	GPL20301	7	4	5.51 ± 0.67	4.04 ± 0.61	3.53	< 0.01
GSE126683	China	GPL16956	3	3	10.23 ± 0.39	7.41 ± 0.39	175.50	< 0.01
GSE13597	UK	GPL96	25	3	6.76 ± 0.58	5.35 ± 0.33	4.07	< 0.01
GSE39826	UK	GPL6244	3	3	8.41 ± 0.07	6.78 ± 0.18	18.25	< 0.01
GSE40290	China	GPL8380	25	8	− 1.80 ± 0.82	− 3.03 ± 1.41	3.06	< 0.01
GSE53819	China	GPL6480	18	18	10.90 ± 0.70	9.79 ± 1.40	3.26	< 0.01
GSE61218	China	GPL19061	10	6	8.08 ± 0.80	6.06 ± 0.61	5.29	< 0.01
In-house IHC	China	–	110	47	6.83 ± 1.5	2.15 ± 1.46	18.01	< 0.01
Total			418	113	–	–		

Mean 1 ± SD1: NPC tissues; Mean0  ± SD0: non-tumor tissues

**Fig. 2:**
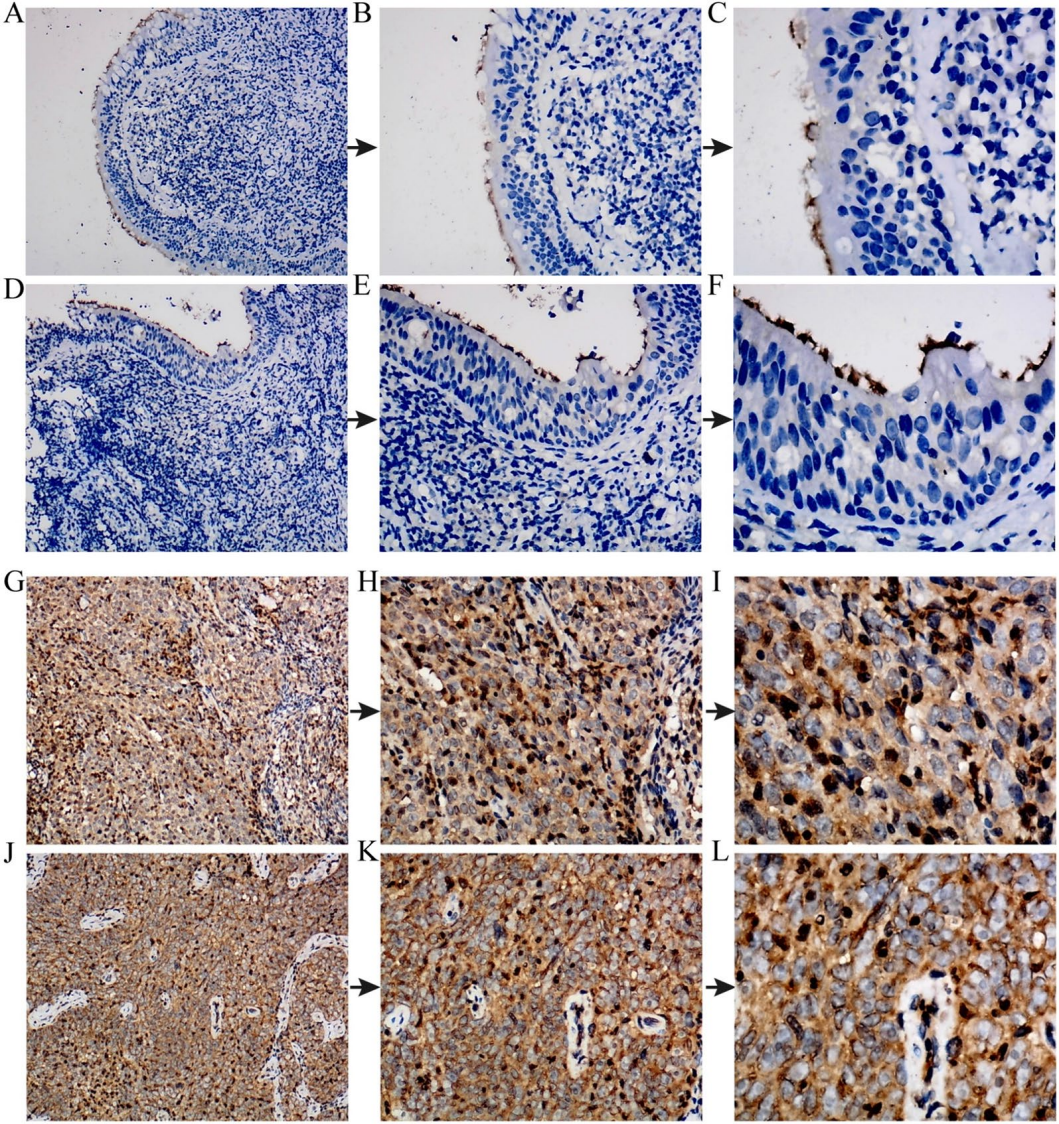
BUB1B protein expression in non-tumor nasopharynx and NPC tissues evaluated by immunohistochemistry. **A**–**F** Non-tumor nasopharynx tissues (magnification × 100 in **A** and **D**, × 200 in **B** and **E**, × 400 in **C** and **F**. **G**–**L** NPC tissues (magnification × 100 in **G** and **J**, × 200 in **H** and **K**, × 400 in **I** and **L**)

**Fig. 3:**
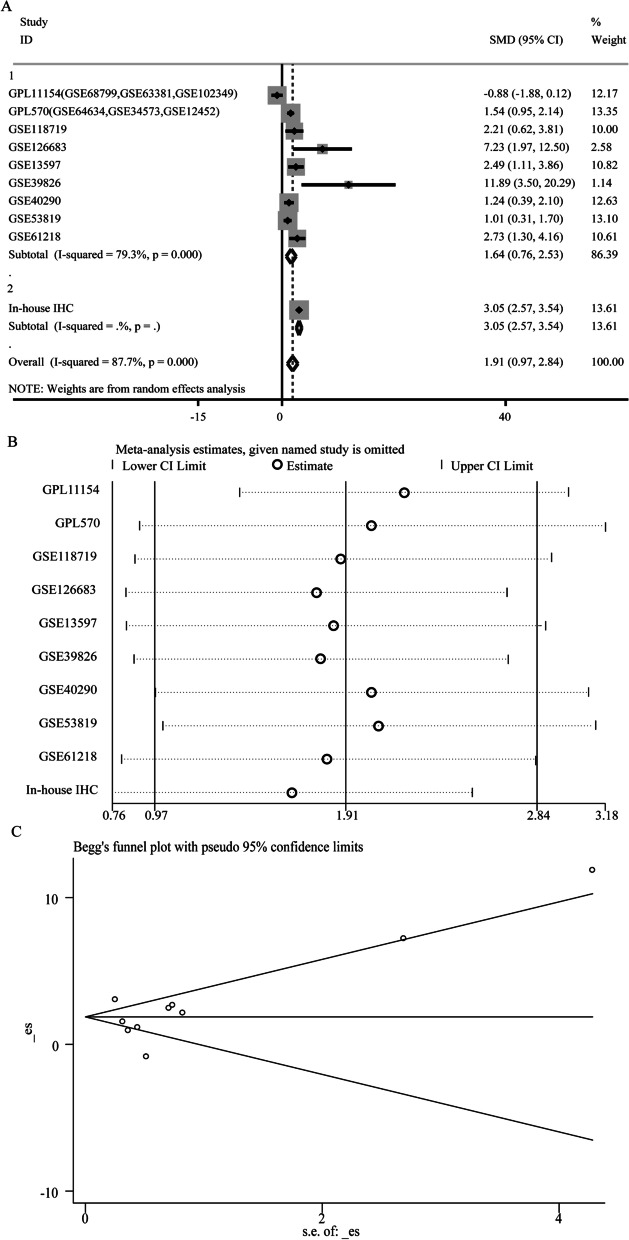
Comprehensive BUB1B expression level in NPC tissues based on multiple cohorts. Results of the forest blot, sensitivity analysis and Begg’s test of BUB1B expression. **A** Forest plot for evaluating BUB1B expression between NPC tissues and non-tumor tissues. **B** Sensitivity analysis of standard mean deviation (random-effects model). **C** Analysis of the detection of publication bias in the comprehensive analysis assessing the expression pattern of BUB1B in NPC.

To validate the results of BUB1B mRNA expression from public gene expression datasets, we assessed the expression of BUB1B proteins in a wide range of NPC tissues and non-NPC tissues to characterize their expressions in situ by IHC (Fig. 2). SMD value of subgroup 2 was 3.054 (95% CI 2.572–3.536, Fig. 3A), which manifested that the expression of BUB1B protein in NPC tissues was remarkably higher than that in non-NPC tissues. This was consistent with the t-test results based on IRS of NPC group and non-tumor group (6.83 ± 1.56 vs. 2.15 ± 1.46, *p* < 0.0001, Fig. 1J). In addition, the ROC indicated that BUB1B protein expression level had a strong ability to distinguish NPC tissues from non-NPC tissues (Fig. 1T).

**Fig. 4:**
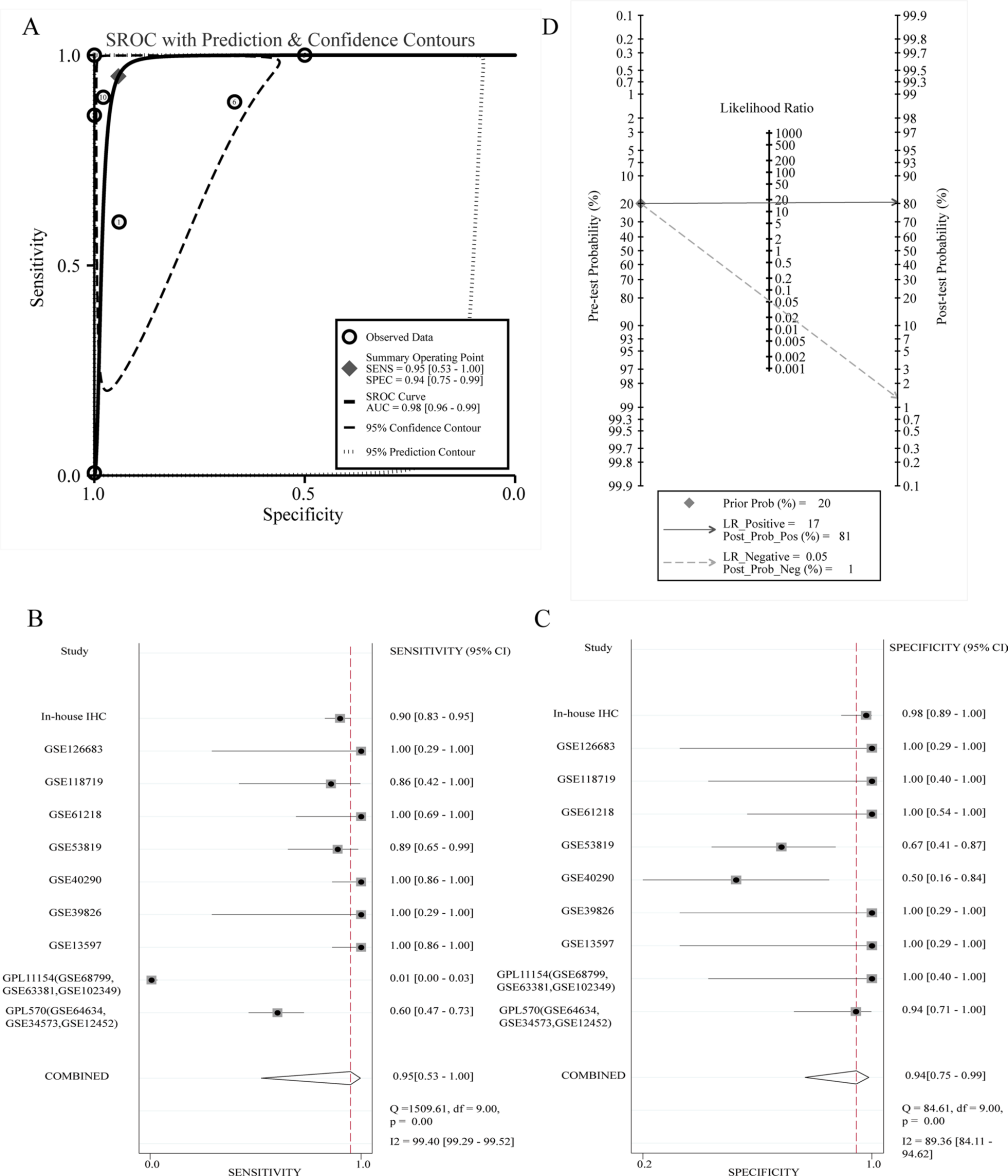
The expression level of BUB1B in NPC tissues assessed by sROC. **A** Summary receiver operating characteristic curve of the distinguishing ability of BUB1B for cancer from non-cancerous tissues. **B** Diagnostic sensitivity analysis. **C** Diagnostic specificity analysis. **D** Fagan’s nomogram

As some single studies were too inadequacy to deduce a reliable conclusion, we integrated the nine gene chips data and our in-house IHC result and performed subgroup analysis. Due to the significant heterogeneity (Overall I^2^ = 87.7%, *p* < 0.001), we adopted a random-effects model. The SMD value of subgroup1 was 1.645 (95% CI 0.764–2.525), confirming that the expression of BUB1B mRNA in NPC tissues was visibly higher than that in non-NPC tissues (Fig. 3A). Sensitivity analysis showed that the included studies could not clearly indicate the source of heterogeneity (Fig. 3B). Begg’ test suggested no significant publication bias (Pr > |z| = 0.245, Fig. 3C). An area under the sROC curve of 0.98 (95% [CI] 0.96–0.99) with sensitivity of 0.95 (95% CI 0.53–1.00) and specificity of 0.94 (95% CI 0.75–0.99) demonstrated strong ability in distinguishing NPC patients from control samples (Fig. 4A–C). In the Fagan plot, with a pre-test probability of 20%, the post-test probability of NPC using BUB1B for a positive test result was 81%, and the probability of a negative test result was 1% (Fig. 4D).

### Relationship between BUB1B and clinical parameters and prognosis of NPC patients

By screening and integrating the datasets with clinical information in the 13 gene chips retrieved above, finally 3 chips (GSE13597, GSE68799, GSE102349) were included in the calculation of staging information, and 3 chips(GSE53819, GSE61218, GSE68799)were included in the age group, gender groups were included in 4 chips (GSE68799, GSE63381, GSE53819, GSE61218). The results showed that the expression of BUB1B was up-regulated in III/IV stage, but there was no significant difference in the expression between different gender or age groups (Additional file 1: Fig. S3A–C). Survival analysis was performed using the survival information of NPC patients in GSE102349. The patients were divided into high group and low group based on the median expression of BUB1B. The analysis results showed that the survival time of patients with higher BUB1B expression was shorter (HR = 7.482, p = 0.0018, Additional file 1: Fig. S3D). Table 2 lists the relationships between BUB1B protein expression and clinical parameters, which illustrated that expression of BUB1B protein of 98 samples from in-house TMAs showed no significant difference in age and gender of NPC patients, which is consistent with external mRNA expression data.

**Table 2:** The relationship between BUB1B protein expression and clinical pathological parameters of in-house samples

Clinicopathological parameters	N	M ± SD	T	*p*
Tissue				
NPC	110	6.83 ± 1.56	− 18.01	< 0.01
Controls	47	2.15 ± 1.46		
Gender				
Male	74	7.04 ± 1.56	− 1.84	0.07
Female	24	6.38 ± 1.50		
Age (year)				
< 60	73	6.93 ± 1.69	0.71	0.48
≥ 60	25	6.72 ± 1.10		

### **Collection of DEGs and BUB1B CEGs**

**Fig. 5:**
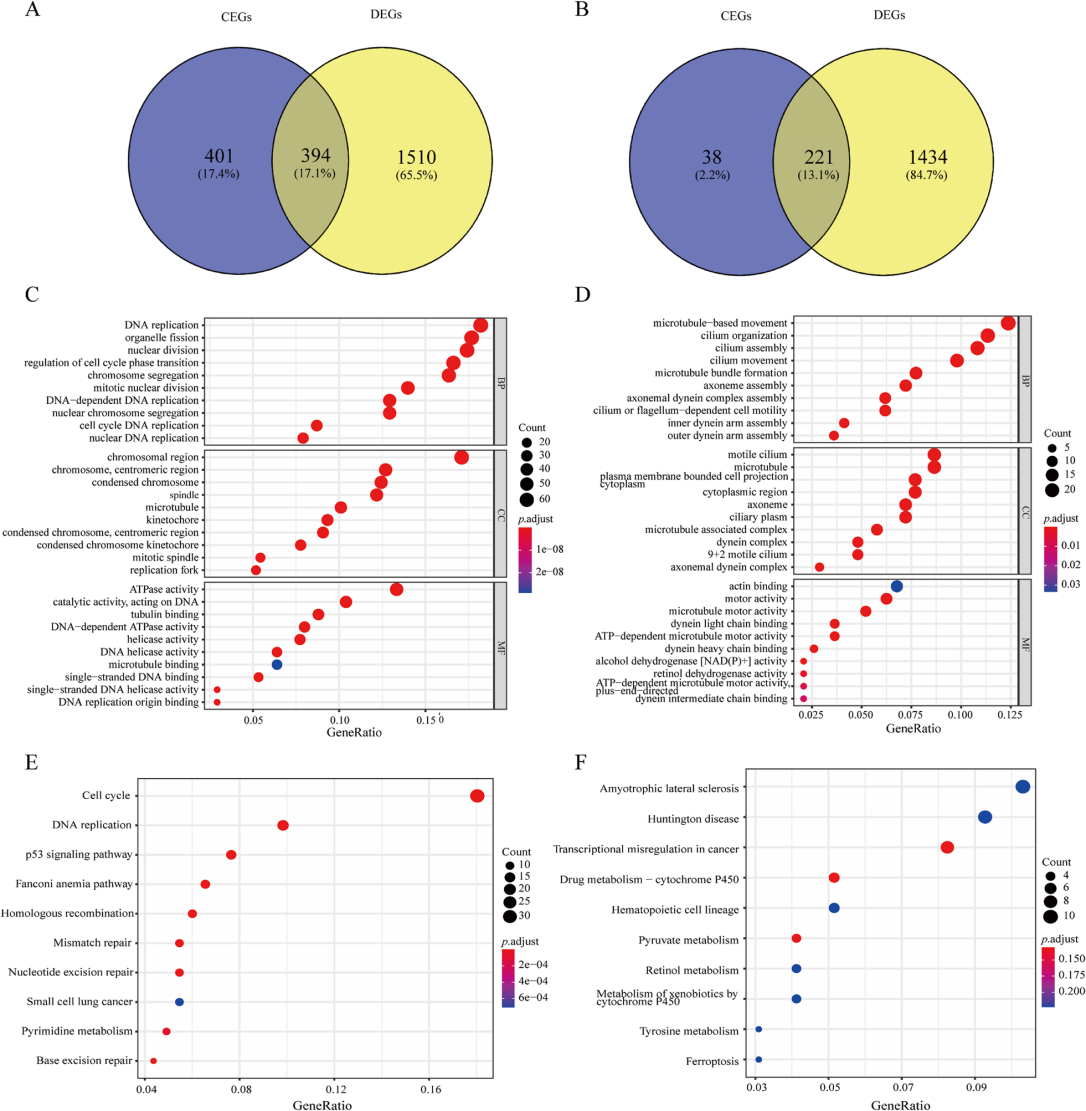
The interaction of CEGs and DEGs of BUB1B and pathways analysis. **A** Venn diagram based on 394 BUB1B positively correlated CEGs and up-regulated DEGs cross genes. **B** Venn diagram based on 221 BUB1B negatively correlated CEGs and down-regulated DEGs cross genes. **C** Gene ontology based on up-regulated DEGs and BUB1B positively correlated CEGs. **D** Gene ontology based on down-regulated DEGs and BUB1B negatively correlated CEGs. **E** Kyoto Encyclopedia of genes and genomes based on up-regulated DEGs and BUB1B positively correlated CEGs. **F** Kyoto Encyclopedia of genes and genomes based on down-regulated DEGs and BUB1B negatively correlated CEGs

After co-expression analysis of BUB1B mRNA expression for each dataset, a total of 795 BUB1B positively correlated CEGs and 259 BUB1B negatively correlated CEGs were screened based on screening standards, and they all appeared in no less than five data sets. In addition, a total of 1655 down-regulated DEGs and 1904 up-regulated DEGs were identified based on 95% CI. A total of 394 BUB1B positively correlated CEGs and up-regulated DEGs cross genes (gene set A, Fig. 5A) and 221 BUB1B negatively correlated CEGs and down-regulated DEGs cross genes (gene set B, Fig. 5B) were obtained.

### **Gene ontology and the “kyoto protocol” gene and genome enrichment analysis based on gene sets A and B**

GO function enrichment analysis was performed to determine the potential molecular mechanisms used by gene set A and B. The first 10 BPs, CCs and MFs of these two gene sets were shown in Fig. 5C, D. In gene set A: the most significant enrichment functions of BPs were DNA replication, organelle fission and nuclear division (*p* < 0.001); regarding CCs, genes were significantly aggregated in chromosomal region, chromosome, centromeric region and condensed chromosome (*p* < 0.001); on the basis of MFs, genes accumulated significantly in ATPase activity, catalytic activity, acting on DNA and tubulin binding (*p* < 0.001, Fig. 5C). In addition, the enrichment analysis of these genes in KEGG showed that they were particularly relevant to Cell cycle and DNA replication (*p* < 0.001, Fig. 5E). In gene set B: for BPs, most significant enrichment function items were microtubule—based movement, cilium organization and cilium assembly (*p* < 0.001); genes markedly assembled at motile cilium, microtubule and plasma membrane bounded cell projection cytoplasm in CCs (*p* < 0.001); on the basis of MFs, genes were significantly accumulated in actin binding, motor activity and microtubule motor activity (*p* < 0.05, Fig. 5D). These genes in KEGG enrichment analysis were revealed to be relevant to the Amyotrophic lateral sclerosis and Huntington’s disease (*p* < 0.05, Fig. 5F).

### **Analysis of the interaction among related core genes of BUB1B in NPC**

The limiting condition of the PPI networks of gene sets A and B was interaction scores greater than 0.4 (Additional file 1: Fig. S4A, B). Nodes that did not interact with other proteins were hidden. Subsequently, we used degree algorithm of cytoHubba to determine the top 10 hub genes of gene set A and top 5 hub genes of gene set B. The hub genes in gene set A were CDC6, MCM2, CDC45, MCM3, CHEK2, CCNB1, MCM7, CDK1, MCM4 and CHEK1, all of which were involved in the cell cycle pathway. The hub genes in gene set B were DNAI2, DNAI1, DNAH1, DNAH9 and DNALI1, all of which were enriched in Huntington’s disease. These relationships between the two hub gene groups were illustrated by PPI network (Additional file 1: Fig. S4C, D).

### **Acquisition of targeted BUB1B upstream regulation factors**

**Fig. 6:**
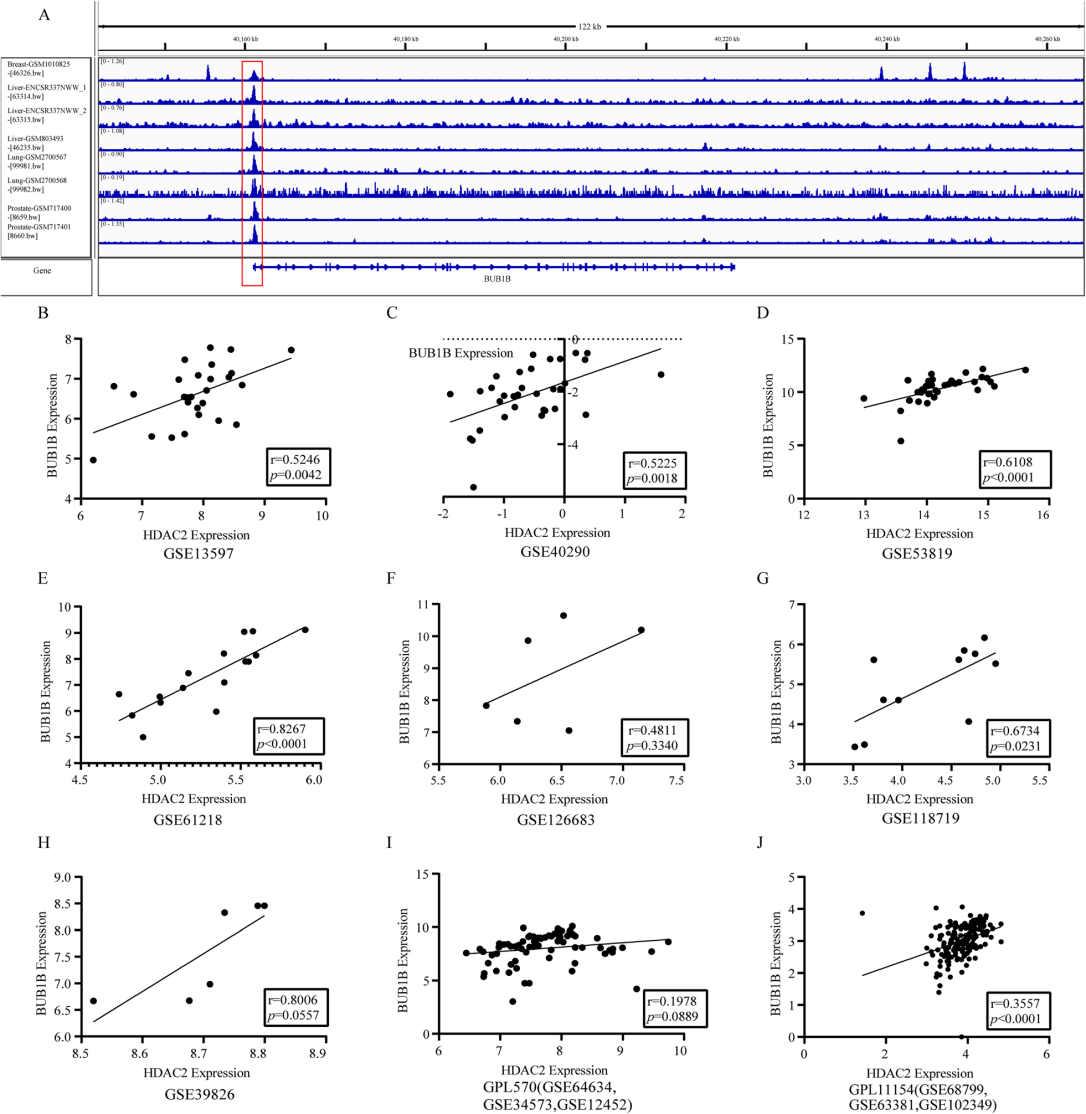
BUB1B could become a potential target of its upstream transcription HDAC2. **A** Chip-seq for the full length of BUB1B is shown. Specific peaks were observed near the promoter region. The promoter region of BUB1B is located from 40,159,069 to 40,161,068, which length is 20 kb. There were specific peaks near the BUB1B promoter region. Some of this information was presented in Table 4. **B**–**J** A correlation analysis of BUB1B and HDAC2 expression level based in 9 data sets

We employed the Cistrome Database toolkit to query the potential regulators of HDAC2 and obtained two hundred results. Part of predicted transcription factors are shown in Table 3. We noticed that HDAC2 not only occupies the forefront of the Regulatory potential (RP) score, but also interacts with BUB1B in the PPI network based on gene set A. In addition, HDAC2 and BUB1B were both enriched in the cell cycle pathway. As Cistrome Data Browser lacks ChIP-seq data related to NPC, we screened the data set which chose epithelial cells as the research object for our study. We collected eight HDAC2 ChIP-seq data sets related to epithelial cells in Cistrome DB and visualized them using IGV (Fig. 6A). In these 8 samples, the BUB1B transcription start site all showed strong signals, indicating that HDAC2 may bind to the BUB1B promoter and promote its transcription. Moreover, we conducted a comprehensive analysis of the expression level of HDAC2 in NPC, and the forest plot and sROC curve both indicated that HDAC2 was highly expressed in NPC (Additional file 1 Fig. S5A, B). Further analysis of the expression correlation between HDAC2 and BUB1B manifested a positive trend in all datasets, and the correlation coefficients were significant in six datasets (Fig. 6B–J). These correlation coefficients were converted to z values ​​and applied for comprehensive analysis (Additional file 1: Fig. S5C). We calculated a combined *r* value of 0.509 (95% CI 0.351 ~ 0.634), indicating that HDAC2 and BUB1B were moderately correlated. In addition, we obtained partial sequences of the BUB1B promoter region from UCSC (Table 4). In summary, we suppose that HDAC2 regulates the expression of BUB1B in NPC to participate in the occurrence and progression of tumor.

**Table 3:** Part of BUB1B upstream transcription factors predicted by Cistrome DB Toolkit

GSM_ID	Factor	Biosource	RP_score
GSM803511	MEF2A	GM12878; B Lymphocyte; Blood	0.575832
GSM1162751	ICE1	HCT-116; Colon	0.569904
GSM2628091	RELB	GM12878; Lymphoblastoid	0.568241
ENCSR338DGO_1	SCRT2	HEK293; Epithelium; Embryonic Kidney	0.564897
GSM1370274	IRF4	OCI-Ly3; B Lymphocyte; Bone Marrow	0.564858
GSM2042854	PPARG	HT29; colorectal adenocarcinoma	0.561362
ENCSR862QUL_2	ZNF382	HEK293; Epithelium; Embryonic Kidney	0.556349
GSM935348	MAX	H1; Embryonic Stem Cell; Embryo	0.5562
GSM935520	MAFF	K562; Erythroblast; Bone Marrow	0.555613
GSM1010893	RUNX3	GM12878; B Lymphocyte; Blood	0.552119
GSM1010825	HDAC2	MCF-7; Epithelium; Breast	0.54982
GSM1010851	SRF	HCT-116; Colon	0.548059
GSM1574277	KLF5	CFPAC-1; Pancreatic ductal	0.538167
GSM518374	NANOG	H1; Embryonic Stem Cell; Embryo	0.534918
GSM777637	NR4A1	K562; Erythroblast; Bone Marrow	0.528851
GSM1010902	SP1	HCT-116; Colon	0.52885
GSM1010756	FOSL1	HCT-116; Colon	0.527778
ENCSR199WXF_2	EED	GM12878; B Lymphocyte; Blood	0.526327
GSM2360622	ZEB1	MDA-231; Epithelium; Mammary Gland	0.525338
GSM1010862	SIN3A	MCF-7; Epithelium; Breast	0.521651

**Table 4:** Basic information for the peak region of BUB1B in Figure 11A.

Transcription factor	Size of sequence	Peak sequence
		gtagcttgcctaaggttgcacatttggtatgattttacagaactagaatc
		cagctttttgaattcaagggtggggcaggaaacagctaggtcagtggcct
		aagaactccggacgggtgagatttggggcagacagcaggggtagtcaccc
		tacaagagtcacgccaagtgcaagcactcagagacatctcccaacactca
		aaacagcaaagaagttctggtgctttaagtgttcctcgctcggctcagag
HDAC2	500 bp	actcggcttcgagccgcgactcaagacagcacctgggggtatttgttttg
		cctaagcctgctgcacttccacggccattgaatcccaaaaactacaattc
		ccattatgcaccgcgagttcgcggactaaacaggcataaactacaagccc
		cagaatgccttgggcgagacgcgagagcacggaggagcggaggggcgtgg
		ccacgtcgaccgcgcgggaccgttaaatttgaaacttggcggctaggggt
		gtagcttgcctaaggttgcacatttggtatgattttacagaactagaatc
		cagctttttgaattcaagggtggggcaggaaacagctaggtcagtggcct
		aagaactccggacgggtgagatttggggcagacagcaggggtagtcaccc
		tacaagagtcacgccaagtgcaagcactcagagacatctcccaacactca
		aaacagcaaagaagttctggtgctttaagtgttcctcgctcggctcagag
		actcggcttcgagccgcgactcaagacagcacctgggggtatttgttttg

## Discussion

The highlight of our study was that it comprehensively demonstrated the up-regulation of BUB1B mRNA in NPC from multiple databases based on 308 NPC samples and 51 controls. Meanwhile, we applied IHC to verify that BUB1B protein expression was also highly expressed in another independent cohort with 110 cases of NPC and 66 cases of non-NPC controls. Furthermore, our study shed light on the underlying upstream regulation factors of BUB1B and its potential molecular mechanisms in NPC.

Before our study, only two studies focused on the role of BUB1B expression levels in NPC, both based on limited sample sizes [11, 12]. The shortcomings of the previous research by Huang et al. [11] and Yue et al. [12] could be solved in our current study. Combining public high throughput data and in-house IHC, we showed the consistent up-regulation of BUB1B in NPC. The case number was 10 folds more than that of Huang et al. with 418 cases of NPC and 113 cases of controls being involved. Various detecting methods being used also enhanced the convincingness of the results, including mRNA and protein levels of BUB1B. Moreover, the samples were from different areas of the world, including China, USA, UK and Singapore. Our study conducted experiments with different detection methods on cases from multiple regions, whose results verified that the expression of BUB1B was up-regulated, suggesting that BUB1B has a certain oncogenic effect in the occurrence of NPC. This effect is suitable for all NPC patients, without regional difference.

To help understand the underlying mechanisms of BUB1B in NPC, the intersecting genes of BUB1B co-expressed genes and DEGs were used to demonstrate the potential signaling pathways related to BUB1B in NPC and to obtain other NPC biomarkers. In the mechanism analysis of this study, BUB1B was positively correlated with the following 10 hub genes involving in the cell cycle pathway: CDC6, MCM2, CDC45, MCM3, CHEK2, CCNB1, MCM7, CDK1, MCM4 and CHEK1. Among them, CHEK1, CDK1, CCNB1, and CDC6 [21–24] in NPC have been studied to some extent. Considering the important role of BUB1B in mitotic checkpoint signaling and chromosome assembly, BUB1B imbalance often leads to aneuploidy and chromosomal instability [25], which may lead to an increase in the incidence of cancer. Consistent with this, the knockdown of BUB1B inhibited brain tumor-initiating cells -driven tumor formation [26]. In addition, the over-expressed BUB1B found in prostate cancer, lung cancer, and breast cancer was associated with the proliferation and metastasis of cancer cells [27–29]. We speculate that the up-regulation of BUB1B expression will promote the expression of the 10 hub genes, thereby accelerating the progress of the cell cycle, and finally enabling the proliferation and invasion of NPC cells.

Interestingly, when predicting the upstream regulatory factor of BUB1B, Histone Deacetylase 2 (HDAC2) was achieved by in-silico method. HDAC2 is a class I isoform of histone deacetylases, which could remove acetyl groups from histones, resulting in a tighter chromatin structure that regulates large amounts of gene transcription [30]. Previous studies shown that HDAC2 was upregulated in breast, colorectal and prostate cancers [31–33], and HDAC2 was associated with cancer-promoting molecular events. In addition, Lee et al. pointed out that HDAC2/3 bound to acetylated BUB1B [34], leading to ubiquitin and degradation of BUB1B to participate in the cell mitosis process, which is consistent with our pathway analysis. However, no researches focused on the regulatory axis of HDAC2-BUB1B in cancer. Our study revealed that HDAC2 was upregulated in NPC, meanwhile, public ChIP-seq data strongly supported the regulatory role of HDAC2 in BUB1B. In conclusion, we hypothesize that the upregulated HDAC2 in NPC induced cell cycle disorders by regulating BUB1B, and ultimately promotes the development of cancer.

Some limitations of this study should not be ignored. First, due to the lack of data, only one dataset was used to evaluate the effect of BUB1B on the prognosis of nasopharyngeal carcinoma patients, which may not be comprehensive. Second, the included data sets were highly heterogeneous, and the influence analysis showed no distinct differences. This may be due to the different study design in the datasets and samples from different countries: China, the United States of America, Singapore and United Kingdom. Third, the precise molecular mechanism of BUB1B in NPC remains to be further studied. Finally, the role of BUB1B in NPC treatment strategies needs to be verified by in vitro and in vivo experiments.

## Conclusion

Our report suggests that up-regulation of BUB1B may promote NPC progression through regulated by upstream factor HDAC2 and interaction with other genes. BUB1B also serves as a potential therapy target in NPC patients. Next, we will conduct a series of in vitro and in vivo experiments to verify this proposition.

## Supplementary Information

**Additional file 1: Fig. S1.** Flow chart of the research design in this investigation. **Fig. S2.** Inclusion and exclusion of datasets. **Fig. S3.** The relationship between BUB1B mRNA expression and clinical parameters and prognosis of NPC patients. The expression of BUB1B mRNA in NPC patients with different stages (A), ages (B) and gender(C) groups. (D) Survival curve of NPC patients based on different groups of BUB1B expression level. **Fig. S4.** PPI network of BUB1B-related genes in NPC. (A)PPI network based on the genes of the first three KEGG pathways of up-regulated DEGs and BUB1B positively correlated CEGs (gene set A). (B) PPI network based on the genes of the first three KEGG pathways of down-regulated DEGs and BUB1B negatively correlated CEGs (gene set B). (C) PPI network based on the hub genes in gene set (A) (D) PPI network based on the hub genes in gene set (B). **Fig. S5.** Comprehensive HDAC2 expression level and comprehensive correlation coefficient in NPC tissues based on nine data sets. (A) Forest plot for assessing HDAC2 expression between NPC tissues and non-tumor tissues. (B) Summary receiver operating characteristic curve of the distinguishing capability of HDAC2 for cancer from non-cancerous tissues. (C) Forest plot for evaluating correlation of HDAC2 and BUB1B expression level.

## Data Availability

The microarray data of GSE68799, GSE63381, GSE102349, GSE64634, GSE34573, GSE12452, GSE118719, GSE126683, GSE13597, GSE39826, GSE40290, GSE53819 and GSE61218 can be acquired from the Gene Expression Omnibus (GEO) database. The ChIP-seq data of GSM1010825, ENCSR337NWW_1, ENCSR337NWW_2, GSM803493, GSM2700567, GSM2700568, GSM717400, GSM717401 can be obtained from Cistrome Data Browser.

## Abbreviations

AUC: Area under the curve
bp: Base pair
BUB1B: BUB1 mitotic checkpoint serine/threonine kinase B
CEGs: Co-expressed genes
CI: Confidence interval
DEGs: Differentially expressed genes
FPKM: Fragments per kilobase of exon model per million reads mapped fragments
GEO: Gene Expression Omnibus
GO: Gene ontology
IGV: Integrative genomics viewer
IHC: immunohistochemical
IRS: Immunoreactivity score
KEGG: Kyoto Encyclopedia of genes and genomes
NPC: Nasopharyngeal carcinoma
PPI: Protein–protein interaction
ROC: Receiver operating characteristic
RP: Regulatory potential
scRNA-seq: Single-cell sequencing RNA technology
SD: Standard deviation
se: Standard error
SMD: Standard mean deviation
SRA: Sequence ReadArchive
sROC: Summary receiver operating characteristic
TCGA: The Cancer Genome Atlas
TMAs: tissue microarrays
TPM: Transcript per million
