# Supplementary material for: Clinical value and potential mechanisms of BUB1B up-regulation in nasopharyngeal carcinoma
Source: BMC Med Genomics. 2022 Dec 28;15:272. doi: 10.1186/s12920-022-01412-8 (PMC9798722; doi:10.1186/s12920-022-01412-8)
Supplement: Supplementary file 1 — Additional file 1: Fig. S1. Flow chart of the research design in this investigation. Fig. S2. Inclusion and exclusion of datasets. Fig. S3. The relationship between BUB1B mRNA expression and clinical parameters and prognosis of NPC patients. The expression of BUB1B mRNA in NPC patients with different stages (A), ages (B) and gender(C) groups. (D) Survival curve of NPC patients based on different groups of BUB1B expression level. Fig. S4. PPI network of BUB1B-related genes in NPC. (A)PPI network based on the genes of the first three KEGG pathways of up-regulated DEGs and BUB1B positively correlated CEGs (gene set A). (B) PPI network based on the genes of the first three KEGG pathways of down-regulated DEGs and BUB1B negatively correlated CEGs (gene set B). (C) PPI network based on the hub genes in gene set (A) (D) PPI network based on the hub genes in gene set (B). Fig. S5. Comprehensive HDAC2 expression level and comprehensive correlation coefficient in NPC tissues based on nine data sets. (A) Forest plot for assessing HDAC2 expression between NPC tissues and non-tumor tissues. (B) Summary receiver operating characteristic curve of the distinguishing capability of HDAC2 for cancer from non-cancerous tissues. (C) Forest plot for evaluating correlation of HDAC2 and BUB1B expression level. [file 12920_2022_1412_MOESM1_ESM.docx]

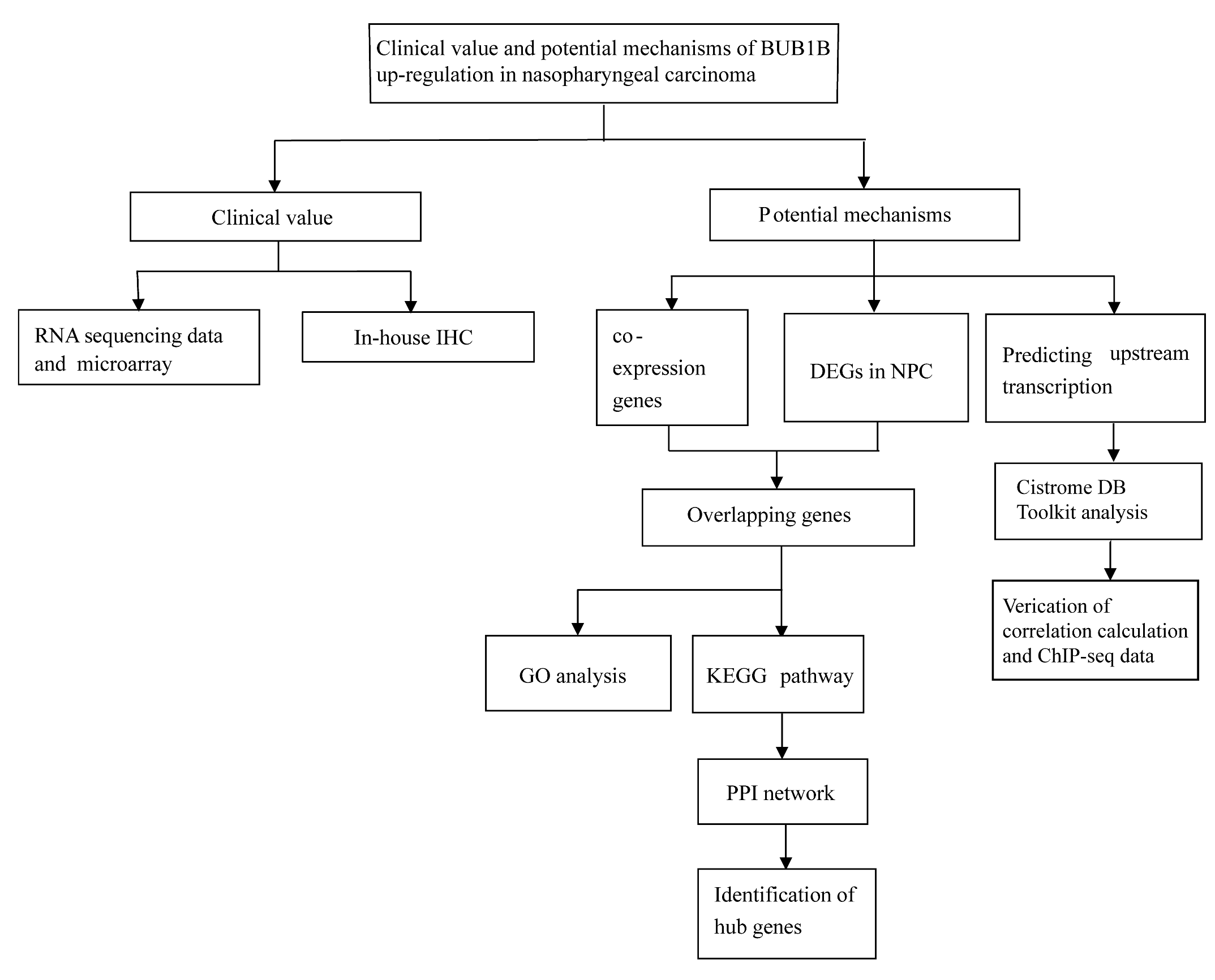


Supplementary Figure 1: Flow chart of the research design in this investigation.


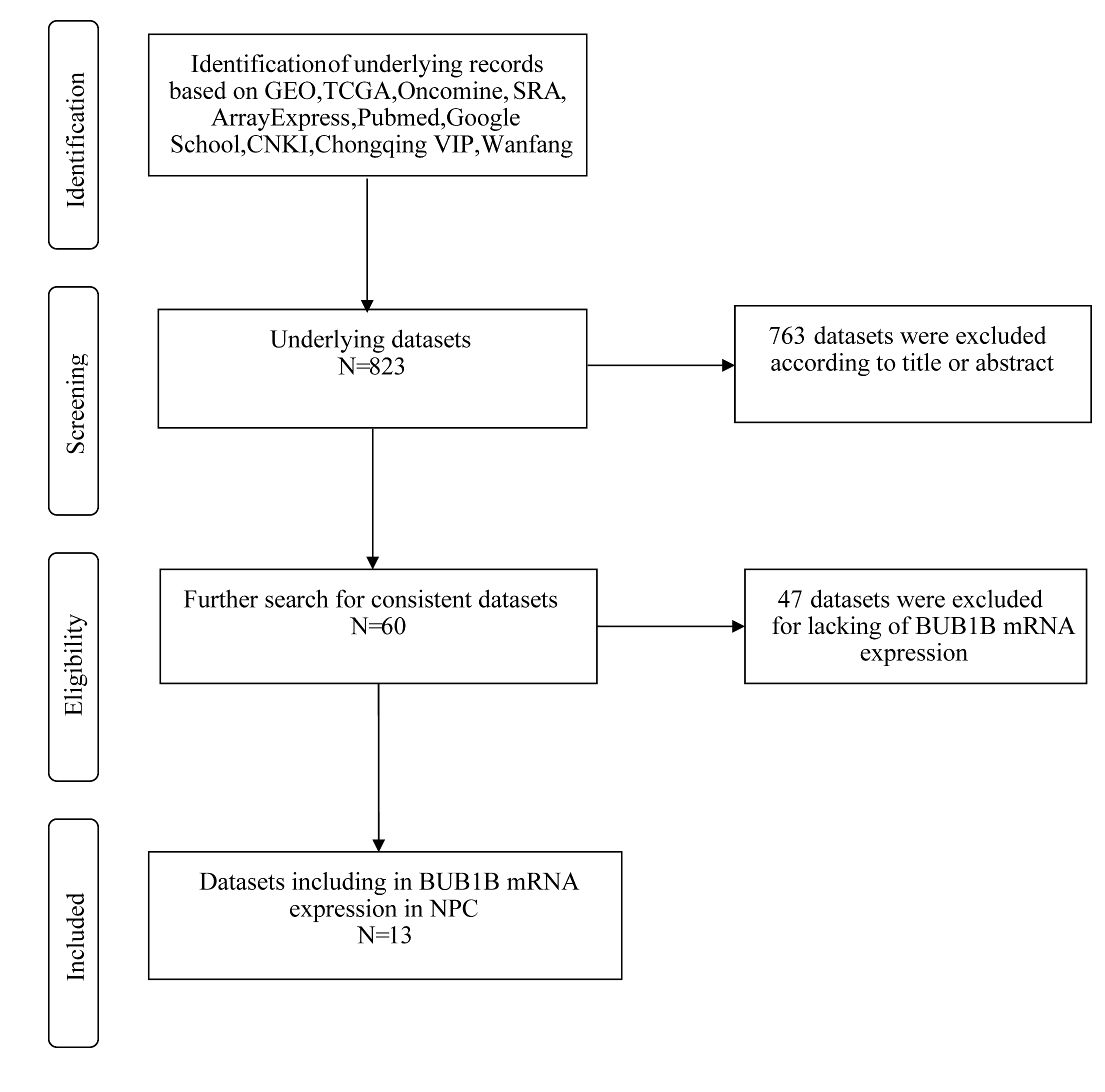


Supplementary Figure 2：Inclusion and exclusion of datasets.


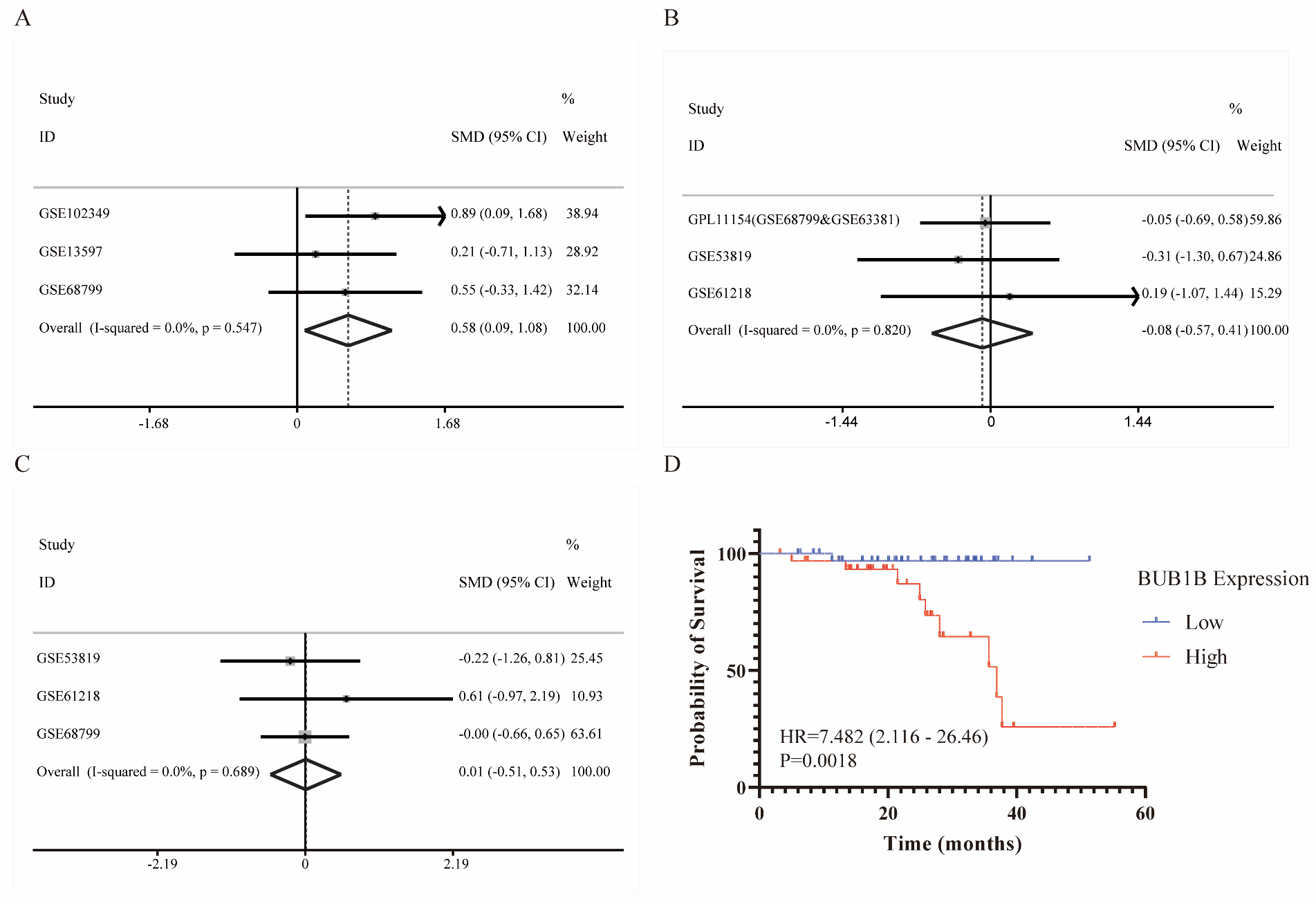


Supplementary Figure 3: The relationship between BUB1B mRNA expression and clinical parameters and prognosis of NPC patients. The expression of BUB1B mRNA in NPC patients with different stages (A), ages (B) and gender(C) groups. (D) Survival curve of NPC patients based on different groups of BUB1B expression level.
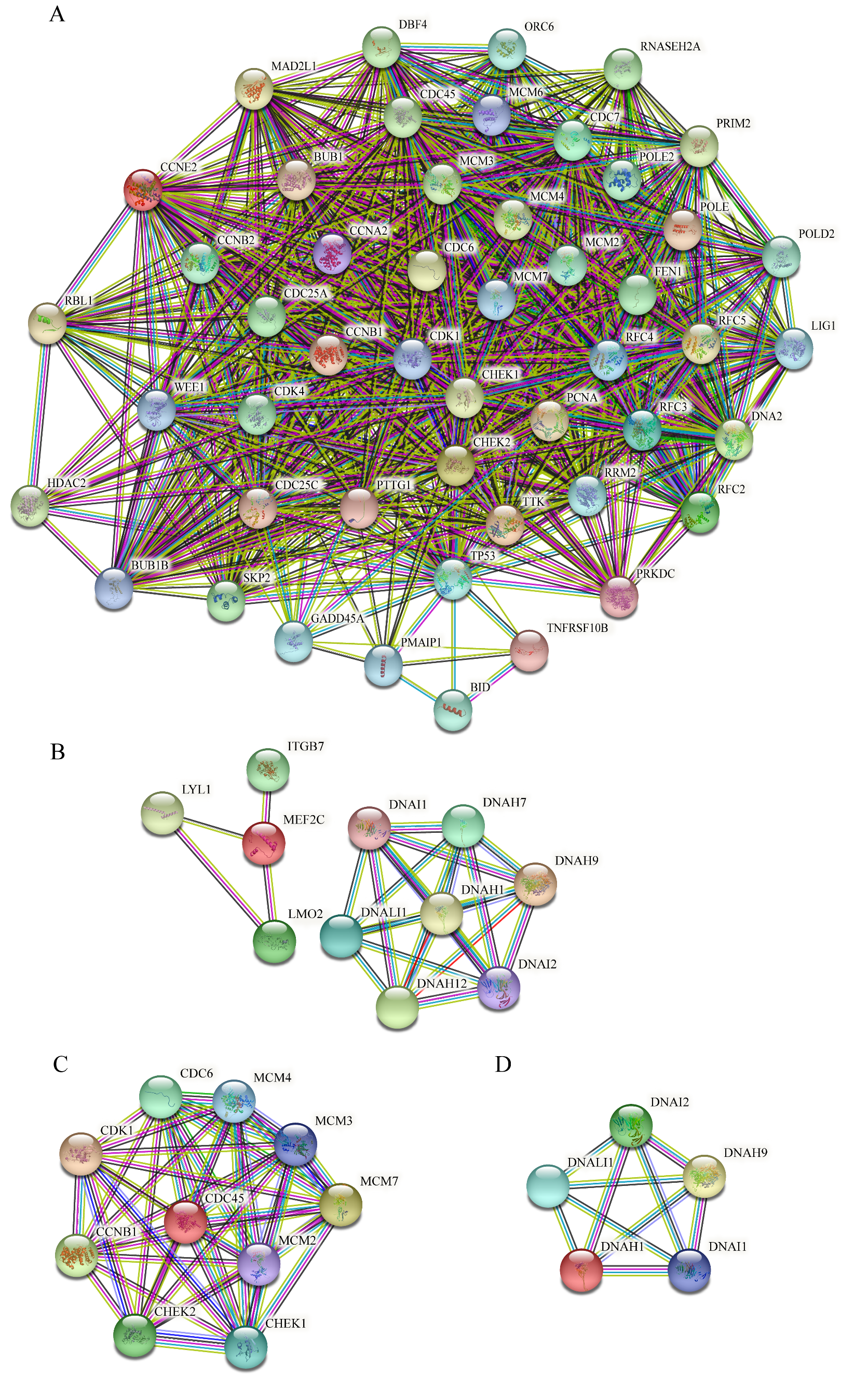


Supplementary Figure 4: PPI network of BUB1B-related genes in NPC. (A)PPI network based on the genes of the first three KEGG pathways of up-regulated DEGs and BUB1B positively correlated CEGs (gene set A). (B) PPI network based on the genes of the first three KEGG pathways of down-regulated DEGs and BUB1B negatively correlated CEGs (gene set B). (C) PPI network based on the hub genes in gene set A. (D) PPI network based on the hub genes in gene set B.


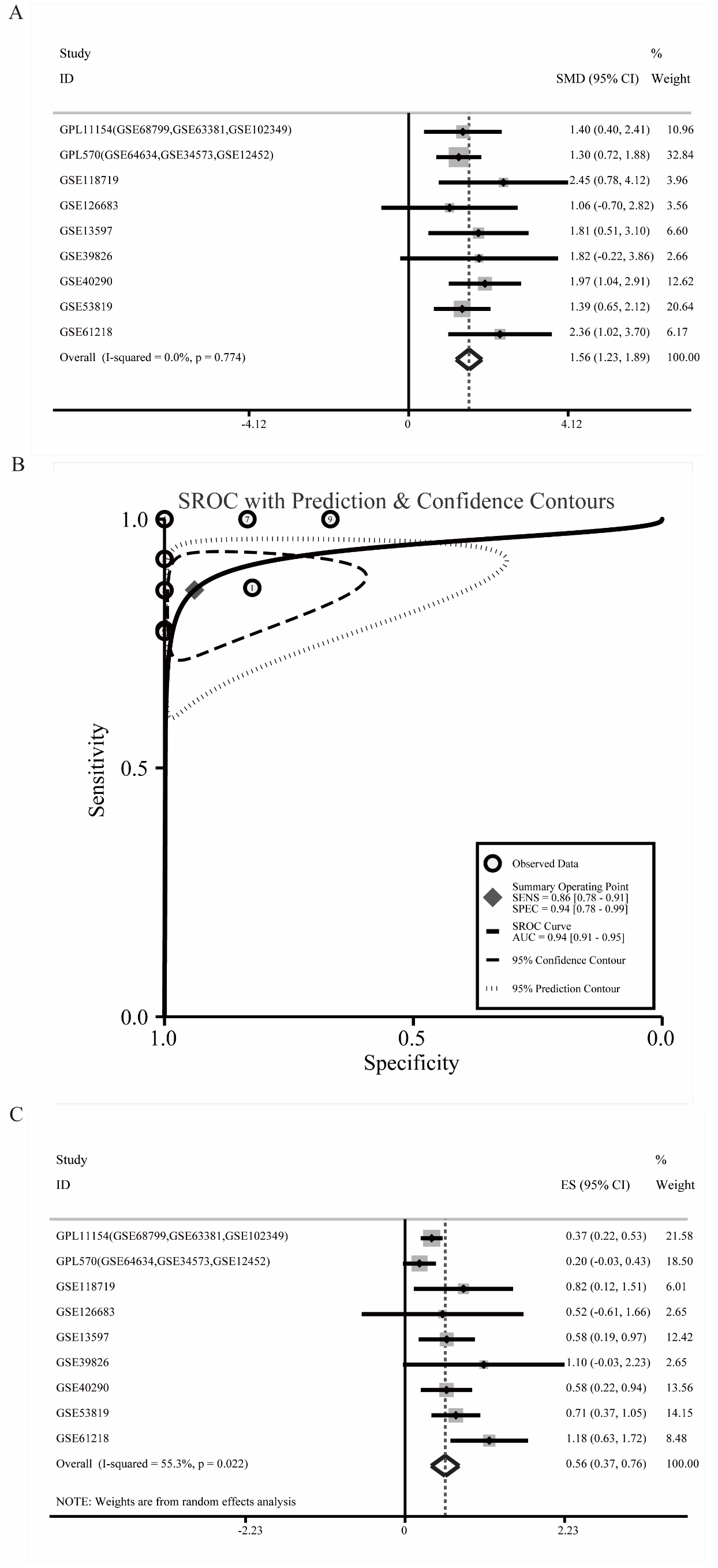


Supplementary Figure 5: Comprehensive HDAC2 expression level and comprehensive correlation coefficient in NPC tissues based on nine data sets. (A)Forest plot for assessing HDAC2 expression between NPC tissues and non-tumor tissues. (B) Summary receiver operating characteristic curve of the distinguishing capability of HDAC2 for cancer from non-cancerous tissues. (C) Forest plot for evaluating correlation of HDAC2 and BUB1B expression level.
